# Open-label, randomized multicentre phase II study to assess the efficacy and tolerability of sunitinib by dose administration regimen (dose modification or dose interruptions) in patients with advanced or metastatic renal cell carcinoma: study protocol of the SURF trial

**DOI:** 10.1186/s13063-018-2613-8

**Published:** 2018-04-12

**Authors:** Guillaume Mouillet, Marie-Justine Paillard, Tristan Maurina, Dewi Vernerey, Thierry Nguyen Tan Hon, Hamadi Almotlak, Ulrich Stein, Fabien Calcagno, Diane Berthod, Elise Robert, Aurelia Meurisse, Antoine Thiery-Vuillemin

**Affiliations:** 1 Department of Medical Oncology, University Hospital of Besançon, 25000 Besancon, France; 2 Methodology and Quality of Life Unit in Oncology, University Hospital of Besançon, 25000 Besancon, France; 3 Université Bourgogne Franche-Comté, INSERM, EFS BFC, UMR1098, Interactions Hôte-Greffon-Tumeur/Ingénierie Cellulaire et Génique, 25000 Besancon, France

**Keywords:** Renal cell carcinoma, Sunitinib, Metastatic, Schedule, Toxicity, Safety, Health-related quality of life

## Abstract

**Background:**

Sunitinib is a tyrosine kinase inhibitor approved in the first-line metastatic renal cell carcinoma (MRCC) setting at the dose of 50 mg daily for 4 weeks followed by a pause of 2 weeks. Due to toxicity, this standard schedule (50 mg daily 4/2) can induce up to 50% of sunitinib dose modification (reduction and/or interruption). The current recommendation in such case is to reduce the dose to 37.5 mg per day (standard schedule 4/2). Recent data highlight an alternative schedule: 2 weeks of treatment followed by 1 week of pause (experimental schedule 2/1). The SURF trial is set up to evaluate prospectively experimental schedule 2/1 when toxicity occurs. This article displays the key elements of the study protocol.

**Methods/design:**

SURF [NCT02689167] is a prospective, randomized, open-label phase IIb study. Patients are included at sunitinib initiation while receiving standard schedule 4/2 (50 mg daily) according to the marketing authorization indication. When a dose adjustment of sunitinib is required, patients are randomized between standard schedule 4/2 (37.5 mg daily) and experimental schedule 2/1 (50 mg daily). Key eligibility criteria are the following: patients with locally advanced inoperable or MRCC who are starting first-line treatment with sunitinib, with histologically or cytologically confirmed renal cancer clear cell variant or with a clear cell component, and with Karnofsky performance status ≥70%. The primary objective is to assess the median duration of sunitinib treatment (DOT) in each group. The key secondary objectives are progression-free survival, overall survival, time to randomization, objective response rate, safety, sunitinib dose intensity, health-related quality of life, and the description of main drivers triggering randomization. We hypothesized that experimental schedule 2/1 would result in an improvement in median DOT from 6 to 8.5 months. It was estimated that 112 patients would be needed in each arm during 24 months. In order to take into account the possibility of treatment discontinuation before randomization, 248 patients are necessary.

**Discussion:**

The SURF trial is asking a pragmatic question adapted to the current practice on what is the best way to adapt sunitinib when treatment-related adverse events occur. The results of the SURF trial will bring high-value data to support the use of an alternative schedule in sunitinib treatment.

**Trial registration:**

ClinicalTrials.gov, NCT02689167. Registered on 26 February 2016.

**Electronic supplementary material:**

The online version of this article (10.1186/s13063-018-2613-8) contains supplementary material, which is available to authorized users.

## Background

Metastatic renal cell carcinoma (MRCC) represents nearly 85% of all kidney cancers. More than 120,000 cases of RCC are being diagnosed each year in Europe and in the USA, and the incidence of RCC seems to be rising.

Sunitinib is an anti-angiogenic tyrosine kinase inhibitor (TKI) indicated for the treatment of advanced or MRCC in adults. It is mainly used in the first-line setting at the initial dose of 50 mg taken orally once daily for 4 consecutive weeks followed by a 2-week rest period to comprise a complete cycle of 6 weeks (schedule 4/2). Recommendations in case of required dose modification indicate a dose reduction to 37.5 mg per day and if necessary further to reduce the dose to 25 mg daily [1]. Another promising adjustment schedule is to continue with the daily dose of 50 mg while making more frequent break periods: 2 weeks of treatment followed by a pause of 1 week (schedule 2/1). This last regimen is expected to reduce adverse events and preserve health-related quality of life (HRQoL) while maintaining the same dose intensity. Maintaining exposure to sunitinib seems essential based on previously reported data linking exposure to drug activity in the MRCC setting [2–4]. The experience of the Cleveland Clinic with 30 patients treated with schedule 2/1 showed a significant improvement in the tolerance for the drug, without grade 4 toxicity and with less than 30% of patients with grade 3 toxicity [5].

In a multicentre one-arm prospective study, 60 patients were treated with 50 mg on schedule 2/1, but it did not result in a lower rate of grade III or higher fatigue and diarrhea when compared to historical data from trials employing schedule 4/2. However, efficacy data showed a robust response rate and a prolonged progression-free survival, suggestive of improved long-term tolerability in patients receiving sunitinib on schedule 2/1 [6]. In another prospective phase II study of an individualized sunitinib schedule, 22 patients got sunitinib 50 mg schedule 2/1 as the optimal schedule with good efficacy on the overall response rate (ORR), progression-free survival (PFS), and overall survival (OS) [7]. Sunitinib administered with schedule 2/1 was associated with less toxicity and higher failure-free survival (FFS) at 6 months than that administered with schedule 4/2, without compromising the efficacy in terms of ORR and time to progression (TTP) in a multicentre, randomized, open-label phase II trial, where 38 patients were treated with schedule 2/1 [8]. Many retrospective studies showed better tolerance and efficacy of schedule 2/1 such as the RAINBOW cohort [9, 10]. The main drawback of these data comes from their retrospective status and the small size of the populations for the prospective trials.

The SURF trial aims at evaluating prospectively at a larger scale the efficacy and safety of sunitinib according to the adaptation of the administration regimen (schedule 4/2 or 2/1) in patients with advanced or MRCC. This article displays the key elements of the study protocol.

## Methods/design

### Study design

SURF is a prospective, multicentre, randomized, open-label study in which patients are randomized between two medication schedules (standard schedule 4/2 vs experimental schedule 2/1) when a dosage adjustment (e.g., adverse event, patient request) of sunitinib is required (Additional file 1). Standard schedule 4/2 is defined as 2 periods comprising 4 weeks “on” with sunitinib followed by 2 weeks “off” without sunitinib. The experimental schedule 2/1 is defined as 2 periods comprising 2 weeks “on” with sunitinib followed by 1 week “off” without sunitinib.

**Fig. 1:**
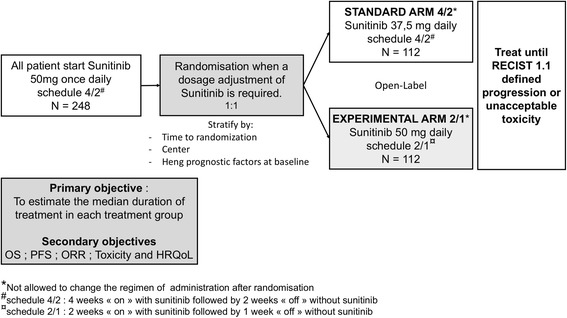
Trial design

At the inclusion, all patients start sunitinib at the recommended schedule 4/2 with sunitinib dosage of 50 mg once daily. When a dosage adjustment of sunitinib is required, patients will be randomized between two arms: one arm in which the dose of sunitinib is reduced to 37.5 mg once daily (standard schedule 4/2, arm 4/2) and one arm in which treatment is administered with a dose maintained at 50 mg once daily (experimental schedule 2/1, arm 2/1) (Fig. 1).

The aim of this randomized clinical trial is to establish whether experimental schedule 2/1 is acceptable in terms of efficacy and toxicity. The main hypothesis is that experimental schedule 2/1 may maintain efficacy in terms of duration of treatment, survival rate, and response rate, has less toxicity, and preserves HRQoL.

### Endpoint definitions

#### Primary endpoint

**Fig. 2:**
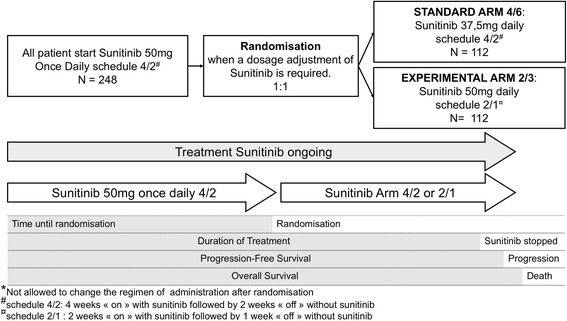
Outcome definition

The primary objective of this study is to estimate the median duration of treatment (DOT) in each treatment group (arm 4/2 vs arm 2/1) calculated from sunitinib initiation (Fig. 2).

#### Secondary endpoints

The key secondary objectives are measured to evaluate the efficacy and the safety of the experimental schedule 2/1 and are the following: are measured to evaluate the efficacy and the safety of experimental schedule 2/1:

- To estimate the PFS and OS of patients in each of the groups and in the overall population included in the study
- To describe the decision factors requiring dose adjustment leading to randomization to the dose of sunitinib
- To estimate the time between the date of randomization and the treatment discontinuation (regardless of the cause) in the two treatment arms
- To estimate the time to randomization defined as the delay between the date of initiation of treatment and the date of randomization
- To evaluate ORR, duration of response, and clinical benefit duration according to RECIST 1.1 criteria [11]
- To describe the toxicity before and after randomization according to NCI CTCAE version 4.0
- To describe dose modifications (dose reduction, interruption, or discontinuation) and to estimate the dose intensity in each treatment group
- To evaluate HRQoL from the initiation of treatment (before, during, and after randomization)
- To estimate time to HRQoL deterioration (TTD) in each of the groups and in the overall population included in the study

### Exploratory objectives

#### The key exploratory objectives are the following:

- To assess the time until the first and second subsequent systemic therapy after study SURF output
- To identify the clinical and biological factors associated with DOT, PFS, and OS
- To identify tissue biomarkers potentially linked to the activity of sunitinib
- To identify blood biomarkers related to the activity and/or toxicity of sunitinib
- To assess the impact of body mass index (BMI) on activity-related parameters of sunitinib

### Characteristics of participants

#### Key inclusion criteria are the following:

- Men or women over 18 years old
- Patients with local, advanced, or inoperable or metastatic RCC who are starting first-line treatment with sunitinib 50 mg (4/2 weeks’ regimen) according to the marketing authorization indication
- Patients with histologically or cytologically confirmed renal cancer clear cell variant or with a clear cell component
- Karnofsky performance status ≥70%
- Adequate organ function:
  - Absolute neutrophil count ≥1500/μL
  - Platelets ≥100,000/μL
  - Hemoglobin ≥10 g/dL
  - Creatinine clearance ≥30 mL/min (by the MDRD formula)
  - Total bilirubin ≤1.5 × ULN (upper limit of the normal range)
  - AST ≤2.5 × ULN and ALT ≤2.5 × ULN or AST and ALT ≤5 × ULN if with liver abnormalities due to liver metastases
- Signature of informed consent for participation

#### Key exclusion criteria are the following:

- Renal carcinoma with no clear cell component
- Previous systemic treatment for RCC regardless of type except immunotherapy (including targeted therapy, chemotherapy, or hormone or experimental therapy). Previous or concomitant treatment with a bisphosphonate or denosumab is allowed
- Patients whose clinical state and comorbidities are not consistent with administration of sunitinib at the initial dose of 50 mg daily with schedule 4/2
- General contraindication, warnings, and precautions to sunitinib treatment as described in the summary of product characteristics [12]
- Major surgery within 4 weeks before sunitinib initiation
- History of symptomatic cerebral metastases, spinal cord compression, or meningeal carcinomatosis. Patients with cerebral metastases discovered incidentally on imaging and who are asymptomatic are not excluded if these metastases have been treated (radiotherapy and/or surgery) with a period of at least 4 weeks between the end of treatment and inclusion into the study and no clinical or radiological signs of relapse, and corticosteroid dose is not exceeding 10 mg/day of prednisone or equivalent. Subjects will be excluded if they have signs of grade ≥2 treatment-related complications
- Any of the following features within 3 months of the administration of sunitinib: myocardial infarction, severe/unstable angina, coronary artery/peripheral artery bypass graft, symptomatic congestive heart failure, cerebrovascular accident, or transient ischemic attack
- Pulmonary embolism or deep vein thrombosis within 3 months of inclusion (unless it is stable, asymptomatic, and treated with a low molecular weight heparin for at least 10 days before inclusion)
- Any known acute or chronic disorder (such as severe chronic obstructive pulmonary disease) which in the opinion of the investigator could impact on the patient’s capacity to receive the study treatment or make interpretation of toxicity or adverse events difficult

### Study procedures and randomization

Patients candidate for first-line treatment with sunitinib and meeting the inclusion/exclusion criteria will be offered participation in the protocol. All the included patients will receive sunitinib 50 mg once daily with schedule 4/2 according to the marketing authorization indication.

When the investigator believes a modification of administration of sunitinib is necessary, the patient will become eligible for randomization. Randomization will take place in all study sites using an IWRS type of centralized system.

Randomization will be performed according to a ratio of 1:1 using minimization technique with stratification according to:

- centre
- Time to randomization: time between initiation of sunitinib and dose adjustment (<6 or ≥6 months)
- Heng prognostic factors at baseline (good and intermediate prognosis groups vs poor prognosis group)

### Clinical measures

**Fig. 3:**
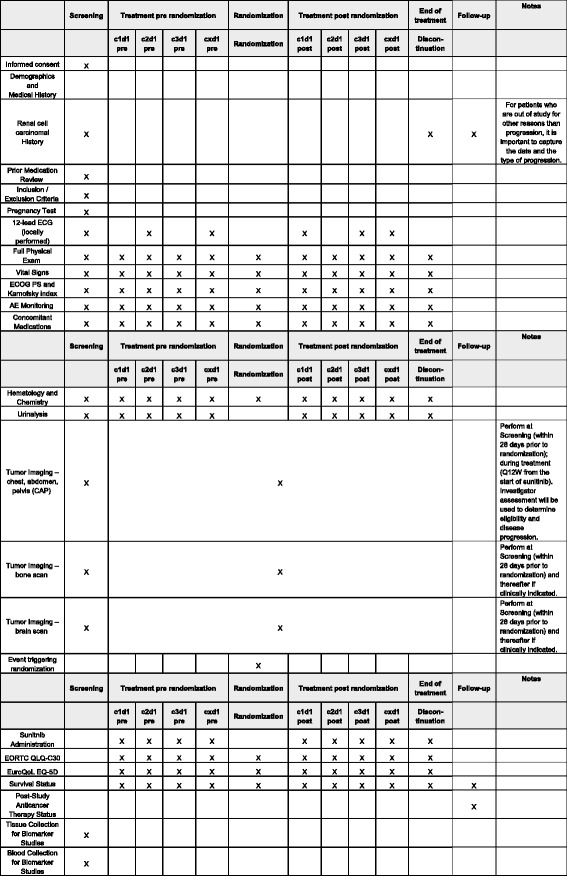
Schedule of enrollment, interventions, and assessments in the SURF study

At treatment initiation (Fig. 3), data collected will include sociodemographic characteristics, oncologic history, symptoms at baseline, biological abnormalities, tumor assessment report, and evaluation of HRQoL (EORTC QLQ-C30 and EuroQoL EQ-5D questionnaires).

During each cycle of sunitinib before and after randomization (every 6 weeks), data collected will include symptoms and biological abnormalities, toxicities according to NCI CTCAE version 4.0, data related to sunitinib (start date, end date, dosage, dose modification and date, monitoring of compliance), toxicities related to sunitinib with grades according to NCI CTCAE version 4.0, and evaluation of HRQoL.

Tumor assessments will be performed every 12 weeks with evaluation of the response by RECIST 1.1 criteria.

During follow-up, cancer treatments provided later (start and end dates, type of treatment, reasons motivating the choice of treatment), the vital status and death-related data (date and cause), and the date and mode of progression for patients who left the study for reasons other than progression will be collected.

### Collection of biological samples

A blood sample of 10 mL will be collected at baseline only if the centre has the necessary equipment for centrifugation and freezing the plasma. The sample will be centrifuged, and the plasma will be frozen on-site within 4 h at −80 °C. The available samples of the primary tumor and metastases will be collected in this study. No additional biopsy will be performed. The parameters evaluated will be based on immunohistochemistry techniques or tumor somatic genomic sequencing.

### Intervention

#### Control arm

In the control arm (arm 4/2), the sunitinib dose is reduced according to the marketing authorization indication: sunitinib 37.5 mg once daily with schedule 4/2. A treatment cycle includes a period of exposure to sunitinib and a pause period for a total of 6 weeks.

#### Experimental arm

In the experimental arm (arm 2/1), the sunitinib dose is maintained at 50 mg once daily (no modification of the dose) with a modification of the drug administration schedule: 2 weeks on treatment followed by a pause of 1 week. A treatment cycle includes two periods of exposure to sunitinib and two pause periods for a total of 6 weeks.

#### Blinding

An identification code is assigned to each subject included in the study. The list of identification of subjects including the code numbers for the names and full addresses of the subjects included in the trial will be retained by the investigator and will not be available for the promoter.

The subjects will be informed that data resulting from the study will be stored in and analyzed by a computer. In particular, it will be made clear that personal data concerning them will be accessible only to the investigator and inspection by official authorities or quality audit by representatives of the promoter of this data is possible.

#### Treatment duration

Patients participate until sunitinib is permanently discontinued for toxicity reason, progression, or death related to renal cancer or not.

The duration of follow-up after discontinuation of sunitinib will be 2 years. All death-related data will be collected as part of the study.

All patients included in this study can leave the study at any time, without having to justify the reason.

Study discontinuation include:

- Subject’s request to stop study treatment (withdrawal of consent)
- Decision of the investigator
- Termination of the study by the sponsor
- Adverse events
- Non-compliance with the protocol
- Protocol-defined disease progression
- Lost to follow-up
- Death

### Follow-up assessments

From treatment initiation, clinical, radiological, and biological data are collected prospectively. Visits are carried out every 6 weeks.

Patients will fill in HRQoL’s questionnaires EORTC QLQ-C30 [13] and EuroQoL EQ-5D [14] every 6 weeks (day 1 of each cycle).

Tumor assessments must be achieved every 12 weeks regardless of treatment cycles. Patients will be monitored for 2 years after discontinuation of sunitinib according to the rhythm of the usual medical care. Any subsequent treatment will be recorded with the date and mode of progression for patients who left the study for reasons other than progression.

### Statistics and data analysis

#### Sample size

The main objective of this randomized phase II trial is to evaluate the median DOT in each arm.

TThe following hypotheses will be tested: H0 (null hypothesis of inefficiency): median DOT of 6 months [15, 16] and H1 (alternative efficiency hypothesis): median DOT of 8.5 months. A Brookmeyer-Crowley type of test will be used to estimate the median. With a one-sided statistical test of significance (*α* 5%) and a power of 80%, it will be required to include 112 patients in each arm for 24 months with at least 12 months of follow-up.

The approximate upper critical value to reject H0 is 7.66 months. The number of patients is increased by 10% to take account of the potential progression or treatment discontinuation by patients before randomization and patients lost to follow-up. It will therefore be necessary to include 248 patients.

### Statistical analysis

All statistical analyses will be carried out in intention-to-treat for effectiveness criteria. Sensitivity analyses will be carried out in per-protocol.

The demographic and baseline data will be described by randomization arm using descriptive statistics.

### Analyses of time-to-event data

The median duration of treatment and other survival rates will be estimated using the Kaplan-Meier method. Median survival will be described with their confidence intervals (one-sided statistical test of significance (*α* 5%)) in each of the arms.

Survival curves will be calculated by the Kaplan-Meier method and described using their medians and their 95% confidence intervals.

Multivariate Cox analysis will be conducted to identify factors potentially influencing survival.

All variables originally collected will be tested by univariate analysis. The variables with *P* ≤ 0.20 will be eligible in multivariate analysis.

No *P* value will be indicated, and the effect size of treatment will be assessed using the HR with its confidence interval.

### Analysis of HRQoL data

Descriptive analysis of HRQoL will be presented at baseline, at randomization, and every 6 weeks until the release of the study. HRQoL data will be resumed across TTD.

TTD is classically defined as the time from inclusion in the study to a first significant deterioration in a HRQoL score as compared to the baseline score with no further significant improvement, or death. Pre-specified targeted HRQoL dimensions will be general health status, physical functioning, fatigue, and pain.

TTD curves will be calculated by the Kaplan-Meier method and described using median and their 99% confidence intervals for the four target dimensions (and 95% confidence intervals for the other dimensions).

#### Analyses of safety

Analyses of toxicities will be performed on all randomized patients who received at least one dose of treatment and analyzed according to the treatment received. The toxicities will be graded according to the NCI CTCAE version 4.0 classification. All toxicities occurring after the first dose of drugs after randomization and after the inclusion will be described according to their grade. The time until toxicity grade 3–4 will be estimated using the Kaplan-Meier method.

### Study duration

Up to 20 French sites will be opened for this study to achieve a total of 248 randomized patients (124 patients in each arm).

The main objective of this study is to estimate the DOT by sunitinib according to the administration regimen. The final analysis of the DOT will take place after 112 events. The total duration of the study (between the first inclusion and the final analysis) is estimated to be 60 months.

## Discussion

At the time this manuscript was written, 80 patients have been included in the SURF trial, which makes it the largest prospective cohort of patients assessing the question of alternative schedule 2/1 of sunitinib. In comparison to the current available data, this trial will be able to provide more robust data about an alternative schedule of sunitinib based on an academic study.

The SURF trial is asking a pragmatic question adapted to the current practice: what is the best way to adapt sunitinib when side effects occur? In the SURF trial, randomization does not occur at the start of sunitinib but when a dose modification is required. The decision to randomize the patient is based on the choice of the physician. The purpose of the SURF trial is to bring robust prospective data of efficacy and side effects that can sustain the use of alternative schedule 2/1 in clinical routine practice. Furthermore, HRQoL data evaluated by the EORTC QLQ-C30 questionnaire will bring patient perspective on the alternative schedule.

One of the limitations of this design is a potential loss of power depending on the number of patients who may leave the study before randomization. We estimated that 10% of the patients could be lost between the initiation of the treatment and a potential randomization.

Recently, the FDA [17] approved sunitinib in an adjuvant setting for high-risk patients following the results of the S-TRAC trial, showing that sunitinib improved PFS in high-risk patients [18]. In this trial, patients started sunitinib at 50 mg with schedule 4/2. When toxicity occurred, dose interruptions or dose reductions to 37.5 mg per day were allowed, depending on the type and severity of the toxicity. An alternative schedule was not allowed. In the sunitinib arm, 48.8% of patients had grade III adverse events and 12.1% grade IV adverse events. Furthermore concerning HRQoL, on most QLQ-C30 subscales, patients in the sunitinib group had lower scores than those in the placebo group. Dose reductions and interruptions because of adverse events occurred in 34.3% and 46.4%, respectively, of the patients in the sunitinib group, and treatment discontinuations owing to adverse events occurred in 86 patients (28.1%) in the sunitinib group. In this setting, improving the management of side effects and maintaining HRQoL will be a high-priority topic.

Therefore, the results of the SURF trial will bring useful data on how to deal with sunitinib administration in case of toxicity, for clinicians dealing with patients having renal cell carcinoma at various stages of the disease.

## Trial status

Date of enrollment of the first participant to the trial: 19 March 2016.

Last protocol version number and date: version 11, 24 November 2017.

Approximate date when recruitment will be completed: March 2018.

## Additional file

Additional file 1: SPIRIT checklist. (DOC 122 kb)

## Abbreviations

BMI: Body mass index
DOT: Duration of treatment
EORTC: European Organisation for Research and Treatment of Cancer
FDA: US Food and Drug Administration
FFS: Failure-free survival
HRQoL: Health-related quality of life
ORR: Overall response rate
OS: Overall survival
PFS: Progression-free survival
QLQ-C30: Quality of Life Core Questionnaire
RCC: Renal cell carcinoma
TKI: Tyrosine kinase inhibitor
TTD: Time to HRQoL deterioration
TTP: Time to progression
